# Advances in Blue Exciplex–Based Organic Light-Emitting Materials and Devices

**DOI:** 10.3389/fchem.2022.952116

**Published:** 2022-07-12

**Authors:** Jie Li, Zhi Li, Hui Liu, Heqi Gong, Jincheng Zhang, Qiang Guo

**Affiliations:** College of Optoelectronic Engineering, Chengdu University of Information Technology, Chengdu, China

**Keywords:** blue exciplex, organic light-emitting diode, cohost, reverse intersystem crossing, thermally activated delayed florescence

## Abstract

Exciplexes possessing thermally activated delayed fluorescence (TADF) characteristics have received much attention in the fields of organic light-emitting materials and devices over the past decade. In general, an exciplex is a physical mixture between a donor (D) with hole transport properties and an acceptor (A) with electron transport characteristics, and the energy difference between the lowest excited singlet state and the lowest excited triplet state is usually fairly small in terms of the long-range charge-transfer process from D to A. In the processes of photoluminescence and electroluminescence, triplet excitons can be converted to singlet excitons through reverse intersystem crossing and then radiate photons to achieve TADF. As a consequence, triplet excitons can be effectively harvested, and the exciton utilization can be significantly enhanced. Up to now, a large number of exciplexes have been developed and applied to organic light-emitting devices. Notably most of them showed green or red emission, while blue exciplexes are relatively few owing to the spectrum characteristics of the large red-shift and broadened emission. In this study, the latest progress of blue exciplex–based organic light-emitting materials and devices is briefly reviewed, and future research is prospected.

## Introduction

Since the realization of the first organic light-emitting diode (OLED) possessing a high brightness of >1,000 cd m^−2^ and a low-driving voltage (*V*_on_) of <10 V (Tang and VanSlyke, 1987), OLEDs based on small molecules (Tang et al., 1989; Adachi et al., 1990), polymers (Burroughes et al., 1990; Peng et al., 1998), and metal–organic complexes (Baldo et al., 1998; Chang et al., 2013) have attracted tremendous attention in the fields of lighting and displays over the past few decades owing to their fascinating merits such as thinness, fast response, and flexibility (Hong et al., 2021). Among these OLEDs, several different kinds of luminescence mechanisms, including traditional fluorescence (Friend et al., 1999; Huang et al., 2012), phosphorescence (Bernhard et al., 2002; Zhou et al., 2014), triplet–triplet annihilation (TTA) (Fukagawa et al., 2012; Jankus et al., 2013), traditional thermally activated delayed fluorescence (TADF) (Endo et al., 2011; Uoyama et al., 2012; Zhang et al., 2012; Li et al., 2013; Li et al., 2021d), hyperfluorescence (Nakanotani et al., 2014; Chan et al., 2021), singlet–triplet inversion (Ehrmaier et al., 2019; Pollice et al., 2021; Li et al., 2022), exciplex-based TADF (Goushi et al., 2012; Li et al., 2014; Oh et al., 2015; Li et al., 2021c; Gu et al., 2022), aggregation-induced emission (AIE)–based TADF (Peng and Shuai, 2021; Suman et al., 2021), and multiple resonance (MR)–based TADF (Lee et al., 2020; Stavrou et al., 2021; Wu et al., 2021; Zou et al., 2022) have been reported. Thus, the exciplex used to be considered an important reason for poor OLED performance, and it thus should be avoided and eliminated (Adachi et al., 1990; Jenekhe, 1995; Morteani et al., 2003). Nevertheless, the studies on enhanced exciplex emission over the past decade suggested the possibility of exciplexes as unique light-emitting materials (Goushi and Adachi, 2012; Sarma and Wong, 2018; Zhang et al., 2021) or highly efficient cohost materials for OLEDs with high efficiencies, low *V*_on_, and low roll-offs (Liu et al., 2015b; Wu et al., 2017; Xiao et al., 2018; Wang et al., 2019; Zhao et al., 2019; Jung and Lee, 2020).

**FIGURE 1:**
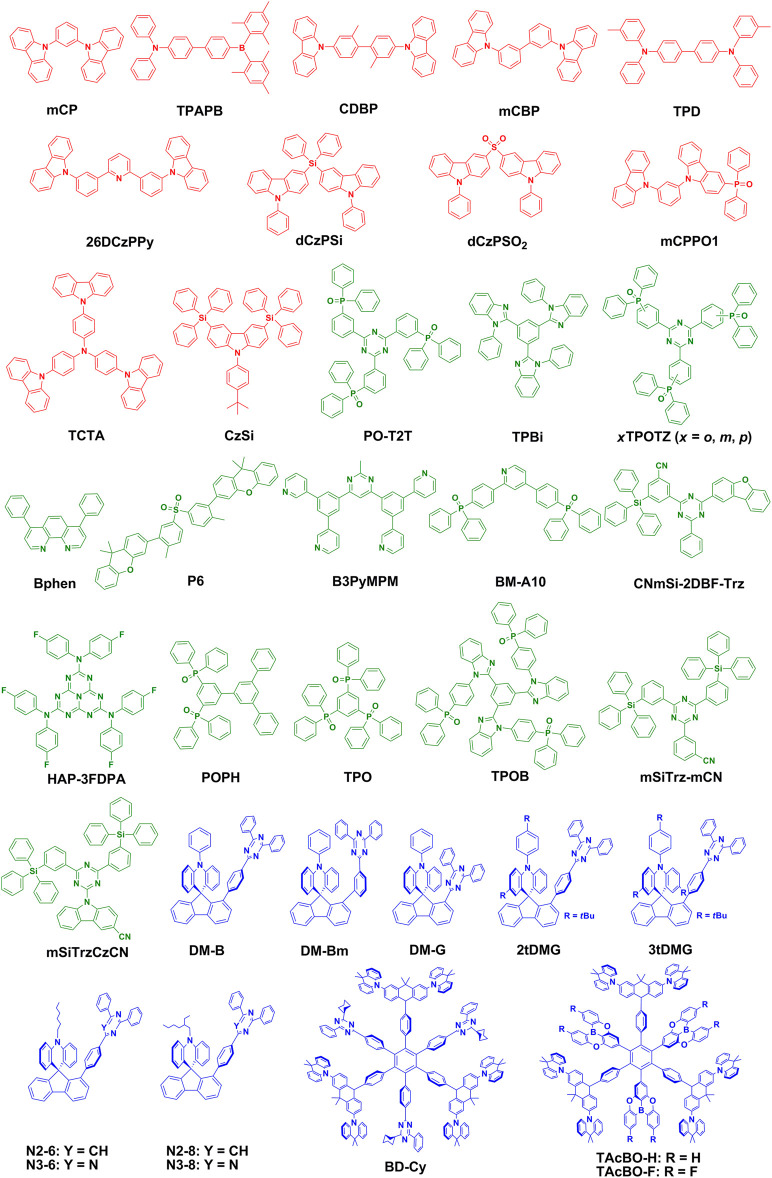
Chemical structures of compounds forming blue exciplexes. The red, green, and blue colors represent donors, acceptors, and intramolecular exciplexes, respectively.

In general, an exciplex originates from the intermolecular charge-transfer (CT)–excited state between the highest occupied molecular orbital of an electron donor (D) and the lowest unoccupied molecular orbital of an electron acceptor (A), leading to a fairly small energy difference (Δ*E*_ST_) between the lowest excited singlet state (S_1_) and the lowest excited triplet state (T_1_) by means of the long-range CT process from D to A (Sarma and Wong, 2018; Shao et al., 2022). In the processes of photoluminescence (PL) and electroluminescence (EL), nonradiative triplet excitons can be converted to be radiative singlet excitons through efficient reverse intersystem crossing (RISC). Thus, triplet excitons can be effectively harvested and the luminescence efficiencies can be significantly enhanced, thus ensuring that exciplexes are an important class of emitters in OLEDs (Hung et al., 2014; Kim and Kim, 2019; Guo et al., 2021). Up to now, a large number of exciplex-based molecular systems have been developed and applied to OLEDs. In particular, most of them are green or red exciplexes, while blue exciplexes are relatively few (Zhang et al., 2021). This is mainly because the realization of blue exciplexes is a herculean task on account of a large red-shifted and broadened exciplex emission spectrum as compared to those of the corresponding D and A compounds (Guo et al., 2021). In this study, the latest progress of blue exciplex–based organic light-emitting materials and devices is briefly reviewed, and future research is prospected. The chemical structures of compounds forming blue exciplexes involved in the following descriptions are depicted in Figure 1, and the EL performance of blue exciplex–based OLEDs is summarized in Table 1.

**TABLE 1:** Electroluminescence performance of blue exciplex–based organic light-emitting diodes.

Exciplex	*λ*_EL_ [nm]	*V*_on_ ^a^ ^)^ [V]	EQE_max_ ^b^ ^)^ [%]	CE_max_ ^c^ ^)^ [cd A^−1^]	PE_max_ ^d^ ^)^ [lm W^−1^]	CIE (x,y)	References
mCP:PO-T2T	471	2.0	8.0	15.5	18.4	(0.17, 0.23)	Hung et al. (2014)
TCTA:Bphen	464	2.6	2.65	3.66	3.82	-	Zhao et al. (2015)
TPAPB:TPBi	468	3.2	7.0 ± 0.4	9.1 ± 0.7	7.2 ± 0.5	(0.14, 0.18)	Chen et al. (2015)
CDBP:PO-T2T	480	2.5	13.0	26.6	27.8	(0.17,0.29)	Liu et al. (2015a)
mCBP:PO-T2T	∼470	-	7.66	15.08	17.78	(0.17, 0.23)	Zhang et al. (2015)
26DCzPPy:PO-T2T	488	3.1	7.8	18.0	17.7	(0.23, 0.36)	Liu et al. (2016)
mCP:*o*TPOTZ	480	3.6	0.4	1.4	1.0	(0.20, 0.30)	Duan et al. (2018)
mCP:*m*TPOTZ	478	2.7	4.34	10.1	11.5	(0.16, 0.29)	Duan et al. (2018)
mCP:*p*TPOTZ	481	2.5	11.1	26.2	32.4	(0.19, 0.36)	Duan et al. (2018)
dCzPSi:PO-T2T	∼480	2.4	8.6	14.7	11.5	(0.15, 0.21)	Hung et al. (2018)
dCzPSO_2_:PO-T2T	∼480	2.4	1.8	4.2	4.8	(0.15, 0.21)	Hung et al. (2018)
CzSi:PO-T2T	465	3.0	6.1	8.9	7	(0.16, 0.21)	Chapran et al. (2019)
mCP:PO-T2T	480	3.0	16.0	27	26.4	(0.16, 0.28)	Chapran et al. (2019)
mCPPO1:PO-T2T	480	3.0	6.5	9.4	8	(0.18, 0.29)	Chapran et al. (2019)
TPD:Bphen	480	3.0	0.46	1.0	0.95	(0.20, 0.31)	Guo et al. (2020)
TCTA:P6	433	6.2	9.1	-	-	-	Tan et al. (2020)
mCP:HAP-3FDPA	437	4.0	10.2	-	-	(0.16, 0.12)	Li et al. (2021b)
DM-B	∼500	2.8	27.4	-	68.1	(0.20, 0.44)	Tang et al. (2020)
DM-Bm	∼500	2.6	21.7	-	62.7	(0.22, 0.48)	Tang et al. (2020)
DM-G	500	3.0	18.5	-	47.5	(0.24, 0.50)	Tang et al. (2020)
2tDMG	504	-	30.8	88.5	71.8	(0.24, 0.53)	Peng et al. (2020)
3tDMG	501	-	20.8	58.0	45.0	(0.24, 0.52)	Peng et al. (2020)
N2-6	480	-	14.1	28.2	14.8	**-**	Wang et al. (2021a)
N2-8	479	-	17.6	34.4	27.0	**-**	Wang et al. (2021a)
N3-6	490	-	14.7	33.4	17.5	**-**	Wang et al. (2021a)
N3-8	488	-	18.9	43.1	27.1	**-**	Wang et al. (2021a)
BD-Cy	477	2.9	18.2	36.8	36.1	(0.18, 0.28)	Wang et al. (2021b)
TAcBO-H	460	3.1	15.8	23.1	-	(0.16, 0.16)	Du et al. (2021)
TAcBO-F	493	3.0	19.5	52.0	-	(0.20, 0.43)	Du et al. (2021)

a Turn-on voltage at 1 cd m^−2^.

b Maximum external quantum efficiency.

c Maximum current efficiency.

d Maximum power efficiency.

## Blue Exciplex–Based Organic Light-Emitting Materials and Devices

Although exciplex emission has been studied for decades, exciplex-based organic light-emitting materials have not received extensive attention for a long time, mainly because of their relatively low luminous efficiency and poor color purity (Gould et al., 1992; Osaheni and Jenekhe, 1994; Gebler et al., 1997; Chao and Chen, 1998; Wang et al., 1998; Cocchi et al., 2002; Kulkarni and Jenekhe, 2008; Zhao et al., 2008; Zhu et al., 2009; Yang et al., 2010). Exciplex-based OLEDs have triggered much attention since Goushi and coworkers demonstrated a pronounced EL enhancement using the RISC process in the 4,4′,4″-tris [3-ethylphenyl (phenyl)amino]triphenylamine (*m*-MTDATA):tris-[3-(3-pyridyl)mesityl]borane (3TPYMB) exciplex system, which showed a relatively high maximum external quantum efficiency (EQE_max_) of 5.4% in view of a rather low photoluminescence quantum efficiency (PLQE) of 26%, exceeding the corresponding limit of fluorescence-based OLEDs (Goushi et al., 2012). Afterward, a much higher EQE_max_ of 10.0% and a maximum power efficiency (PE_max_) of 47.0 lm W^−1^ were achieved by changing the exciplex system from *m*-MTDATA:3TPYMB to *m*-MTDATA:2,8-bis(diphenylphosphoryl)dibenzo [*b*,*d*]thiophene (PPT) (Goushi and Adachi, 2012).

Since then, exciplex-based emitters have aroused widespread attention by virtue of their fascinating optoelectronic properties, and a number of efficient blue exciplex–based OLEDs have been realized (Shao et al., 2022). In 2014, Hung and coworkers attained a record-high blue exciplex OLED (*λ*_EL_ = 471 nm) with an EQE_max_ of 8.0% based on 1,3-bis(*N*-carbazolyl)benzene (mCP):2,4,6-tris[3-(1*H*-pyrazol-1-yl)phenyl]-1,3,5-triazine (PO-T2T) (Hung et al., 2014). In 2015, Zhao et al. (2015) achieved a blue exciplex–based OLED based on 4,4′,4″-tri (*N*-carbazolyl)triphenylamine (TCTA):4,7-di-phenyl-1,10-phenanthroline (Bphen), which displayed a low EQE_max_ of 2.65%. Chen et al. (2015) developed a highly efficient blue exciplex system using a novel electron donor molecule (4-dimesitylboryl)phenyltriphenylamine (TPAPB) and a conventional electron acceptor 1,3,5-tris(1-phenyl-1*H*-benzimidazol-2-yl)benzene (TPBi), while a blue-emitting device containing TPAPB:TPBi exhibited a low *V*_on_ of 3.2 V, a high EQE_max_ of 7.0 ± 0.4%, and CIE coordinates of (0.14, 0.18). Liu and coworkers reported an efficient blue exciplex emitter consisting of 4,4′-bis(9-carbazolyl)-2,2′-dimethylbiphenyl (CDBP):PO-T2T, which showed obvious TADF emission and intrinsically high T_1_, making itself an excellent candidate as a blue emitter or a host for green and red phosphors (Liu et al., 2015a). Meanwhile, the CDBP:PO-T2T exciplex–based blue device delivered a record-high EQE of 13.0% with an EL emission peak at 480 nm and CIE coordinates of (0.17, 0.29). Zhang et al. (2015) demonstrated that the energy could transfer from blue exciplexes to both fluorescent and phosphorescent orange dopants using an efficient blue exciplex system of 9,9′-biphenyl-3,3′-diylbis-9*H*-carbazole (mCBP) and PO-T2T as the electron donor and acceptor, respectively, and a high EQE_max_ of 7.66% with CIE coordinates of (0.17, 0.23) were realized. Liu et al. (2016) investigated the EL property of a blue exciplex of 2,6-bis[3-(9H-carbazol-9-yl)phenyl]pyridine (26DCzPPy):POT2T and an OLED incorporating this exciplex as an emitting layer turned on at 3.1 V, and realized a high EQE_max_ of 7.8% with blue emission peaked at 488 nm. Based on the acceptors *x*TPOTZ (*x* = *o*, *m*, or *p*), which are triphenyltrazine derivatives substituted with diphenylphosphine oxide groups at *ortho-*, *meta-*, and *para-*positions, respectively (Jia et al., 2015), Duan et al. (2018) constructed a series of exciplexes mCP:*x*TPOTZ (*x* = *o*, *m*, or *p*). Thus, in virtue of the strongest electron-withdrawing effect and appropriate steric hindrance, an efficient sky-blue OLED based on mCP:*p*TPOTZ realized an ultralow *V*_on_ of 2.5 eV, a high CE_max_ up to 26.2 cd A^−1^, a high PE_max_ of 32.4 lm W^−1^, and an EQE_max_ of 11.1%. Hung et al. (2018) designed two new nonconjugated linked dicarbazole materials, diphenylbis(9-phenyl-9*H*-carbazol-3-yl)silane (dCzPSi) and 3,3′-sulfonylbis(9-phenyl-9*H*-carbazole) (dCzPSO_2_). The electron-transporting acceptor, PO-T2T, was introduced to give two exciplex-forming systems, dCzPSi:PO-T2T and dCzPSO_2_:PO-T2T. The dCzPSi:PO-T2T–based device revealed a *V*_on_ as low as 2.4 V and a high EQE_max_ of 8.6% with the CIE coordinates of (0.15, 0.21), significantly higher than that of the device based on dCzPSO_2_:PO-T2T (EQE_max_ = 1.8%), which is due to the fact that dCzPSO_2_:PO-T2T possessing a large Δ*E*_ST_ is unfavorable in forming a CT complex.

In 2019, Chapran and coworkers investigated the exciplex properties by selecting PO-T2T as an electron acceptor with different electron donors, and the emissions of these exciplexes spanned from blue to orange-red regions (Chapran et al., 2019). The blue-emitting exciplexes CzSi:PO-T2T–, mCP:PO-T2T–, and mCPPO1:PO-T2T–based OLEDs exhibited high EQE_max_ of 6.1%, 16.0%, and 6.5%, respectively. Guo et al. (2020) reported a blue exciplex–based OLED based on *N*,*N*′-bis(3-methylphenyl)-*N*,*N*′-diphenylbenzidine (TPD):Bphen, which exhibited a relatively low EQE_max_ of 0.46%, possibly resulting from the low PLQE of the exciplex. Tan et al. (2020) designed and investigated a series of donor–acceptor–donor materials based on sulfone substituents as acceptors and found that one of these materials (P6) could form blue exciplexes with TCTA. The TCTA:P6-exciplex–based OLED showed a high EQE_max_ of 9.1% and deep-blue emission with *λ*_EL_ = 433 nm. Li et al. (2021b) designed and synthesized a heptazine-based electron acceptor, 2,5,8-tris[di (4-fluorophenyl)amine]-1,3,4,6,7,9,9b-heptaazaphenalene (HAP-3FDPA), and the exciplex system of 8 wt% mCP:HAP-3FDPA exhibited a high PLQE of 53.2%. More importantly, an OLED containing this exciplex system as an emitting layer showed deep-blue emission with CIE coordinates of (0.16, 0.12), and a rather high EQE_max_ of 10.2% along with a low roll-off.

Of late, intramolecular exciplexes based on through-space charge transfer (TSCT) are an attractive class of emitters with spatial D/A architecture and TADF characteristics (Shao and Wang, 2020; Li B. et al., 2021; Xue and Xie, 2021). For intramolecular exciplexes, the D and A segments are spatially proximate to each other but are physically separated by a nonconjugated structure (Shao and Wang, 2020; Yang et al., 2020). Tang and coworkers presented an intramolecular design strategy for exciplex-based emitters via a space-confined CT to enhance the light emission (Tang et al., 2020). By connecting the donor and acceptor units *via* a rigid linker, the electronic coupling between the donor and acceptor units is sufficient to allow for efficient direct absorption by the CT state. In contrast to more flexible or less strongly coupled samples, the three rigid sky-blue exciplex emitters, 1′-[4-(4,6-diphenyl-1,3,5-triazin-2-yl)phenyl]-10-phenyl-10*H*-spiro[acridine-9,9′-fluorene] (DM-B), 1′-[3-(4,6-diphenyl-1,3,5-triazin-2-yl)phenyl]-10-phenyl-10*H*-spiro[acridine-9,9′-fluorene] (DM-Bm) and 1′-(4,6-diphenyl-1,3,5-triazin-2-yl)-10-phenyl-10*H*-spiro[acridine-9,9′-fluorene] (DM-G) possess very high PLQEs of >90% when incorporated in a solid matrix. The sky-blue OLEDs based on DM-B achieved a fairly high EQE_max_ of 27.4% along with a small efficiency roll-off. Peng et al. (2020) developed two greenish-blue TADF emitters with a tilted face-to-face alignment of D/A units presenting intramolecular noncovalent interactions, 2-(tert-butyl)-10-[4-(tert-butyl)phenyl]-1′-[4-(4,6-diphenyl-1,3,5-triazin-2-yl)phenyl]-10*H*-spiro[acridine-9,9′-fluorene] (2tDMG) and 2,7-di-tert-butyl-10-[4-(tert-butyl)phenyl]-1′-[4-(4,6-diphenyl-1,3,5-triazin-2-yl)phenyl]-10*H*-spiro[acridine-9,9′-fluorene] (3tDMG). 2tDMG and 3tDMG achieved extremely high EQE_max_ of 30.8% (evaporation-process) and 20.2% (solution-process), respectively. These excellent results opened new avenues for the study of spatial electronic interactions in organic light-emitting materials. Wang T. T. et al. (2021) designed and synthesized four small blue-emitting molecules containing a spiro-scaffold based on fluorene, namely, N2-6, N2-8, N3-6, and N3-8, while the rather short D–A distance led to large steric hindrance as well as a π-stacking manner, favoring CT from D to A. The blue OLED based on N3-8 achieved a high EQE_max_ of 18.9%. Wang and coworkers reported the design of π-stacked dendrimers consisting of cofacially aligned D and A for highly efficient OLEDs, and the dendritic structure and orthogonal configuration led to the TSCT emission (Wang X. et al., 2021). Thus, the blue device based on the dendrimer BD-Cy showed promising performance with CE_max_ = 36.8 cd A^−1^, EQE_max_ of 18.2%, and PE_max_ = 36.1 lm W^−1^. Du et al. (2021) reported two blue TSCT dendrimers consisting of dendritic triacridan donors and oxygen-bridged triarylboron acceptors, TAcBO-H and TAcBO-F. More importantly, the solution-processed OLEDs based on these two dendrimers exhibited blue EL emission and a high EQE_max_ of >15%.

## Blue Exciplexes as Cohost Materials in Blue Phosphorescent Organic Light-Emitting Diodes

Balanced carrier transporting for electron and hole in the emitting layer is significant for the OLED performance in the EL process. To achieve good electron and hole balance, various host materials possessing bipolar characteristics have been developed. There are two approaches to realize bipolar hosts. One is to design single molecules consisting of both hole and electron-transporting units (Su et al., 2008; Lee et al., 2009; Chou and Cheng, 2010). The other is to use exciplex-based cohosts inherently containing hole and electron-transporting molecules (Park et al., 2013a; Park et al., 2013b; Lee et al., 2013). In particular, the latter approach commonly does not require a new molecular synthesis, and the hole and electron mobilities of the exciplex can be tuned by adjusting the ratio of hole and electron-transporting molecules. Thus, exciplex-based cohost materials are conductive to the achievement of OLEDs with low *V*_on_ and high efficiencies (Park et al., 2013b; Lee et al., 2013). To realize high-performance blue phosphorescent OLEDs (PhOLEDs) utilizing exciplex-based cohost materials, the T_1_ level of the exciplex should be lower than those of the consisting molecules in order to confine the excitation energy in the exciplex state, not to transfer to the D and A molecules. Meanwhile, the T_1_ level of the exciplex should also be higher than that of a phosphorescent dopant to guarantee the energy transfer from the exciplex to a blue dopant. Hence, it seems to be a challenging issue to attain an ideal exciplex system meeting these requirements (Jung and Lee, 2020; Tan et al., 2020).

In 2014, Shin et al. (2014) reported a high efficiency blue-emitting PhOLED approaching the theoretical efficiency limit (EQE_max_ = 29.5%) using a blue exciplex cohost of mCP:bis-4,6-(3,5-di-3-pyridylphenyl)-2-methylpyrimi-dine (B3PYMPM) and a phosphorescent emitter of iridium (III)bis[(4,6-difluorophenyl)-pyridinato-N,C2′]picolinate (FIrpic), and meanwhile, the OLED exhibited a low *V*_on_ of 3 V and low-efficiency roll-off. In 2015, Ban and coworkers designed and synthesized a novel electron-transporting molecule (5-terphenyl-1,3-phenylene)bis(diphenylphosphine oxide) (POPH), and the solution-processed blue PhOLED incorporating a blue exciplex–based cohost TCTA:POPH displayed an extremely low *V*_on_ of 2.7 V, a high PE_max_ of 22.5 lm W^−1^, and a very low-efficiency roll-off even the luminance was up to 10,000 cd m^−1^ (Ban et al., 2015). Lee et al. (2015) reported an efficient exciplex–based cohost system of mCP:PO-T2T, and a high-performance blue PhOLED using the exciplex cohost doped with FIrpic possessing a remarkably high EQE_max_ of 30.3%, PE_max_ of 66 lm W^−1^, and a low *V*_on_ of 2.4 V was realized. Based on the time resolved PL measurement, these results should be ascribed to the suitable T_1_ level of the exciplex (2.64 eV) ,which is lower than the T_1_ levels of the consisting molecules of mCP (2.94 eV) and PO-T2T (2.99 eV), and higher than that of FIrpic (2.63 eV), so that the exciplex system well confines the excitons in the exciplex state, followed by energy transfer to a blue dopant of FIrpic. In 2016, Ban et al. realized a highly efficient blue PhOLED based on a blue exciplex cohost system of TCTA:1,3,5-tris(diphenylphosphoryl)benzene (TPO) with the CE_max_ of 23.8 cd A^−1^ and PE_max_ of 15.8 lm W^−1^ (Ban X. X. et al., 2016). Afterward, they designed and synthesized a novel electron acceptor 1,3,5-tris(1-[4-(diphenylphosphoryl)phenyl]-1*H*-benzo [d]imidazol-2-yl)benzene (TPOB) to form an exciplex-type cohost with TCTA, and the solution-processed blue PhOLED achieved an extremely low *V*_on_ of 2.8 V and a high PE_max_ of 22 lm W^−1^ along with a low-efficiency roll-off (Ban X. et al., 2016). Lim et al. (2017) developed an exciplex-forming cohost composed of mCP as the donor and 2,4-bis[4-(diphenylphosphoryl)phenyl]pyridine (BM-A10) as the acceptor for deep-blue PhOLEDs achieving a *V*_on_ of 2.9 eV, a rather high EQE_max_ of 24% with CIE coordinates of (0.15, 0.21). Yun and coworkers designed n-type molecules with isomeric molecular structure, while the corresponding exciplex cohosts formed by mCBP:3-(4,6-bis[3-(triphenylsilyl)phenyl]-1,3,5-triazin-2-yl)benzonitrile (mSiTrz-mCN) showed blue emission (Yun et al., 2021a). The deep-blue PhOLED employing this exciplex as a cohost showed a low *V*_on_ of 2.8 V and a high EQE_max_ of 21.0% with a color coordinate of (0.14, 0.18). Afterward, Yun et al. developed a bipolar n-type host material, 9-(4,6-bis[3-(triphenylsilyl)phenyl]-1,3,5-triazin-2-yl)-9*H*-carbazole-3-carbonitrile (mSiTrzCzCN), and the blue-emitting mCBP:mSiTrzCzCN exciplex system showed a high T_1_ energy close to 3.0 eV (Yun et al., 2021b). The mCBP:mSiTrzCzCN exciplex–based deep-blue PhOLED realized a high EQE_max_ of 21.8% and a lifetime elongation of more than double relative to the conventional n-type host-based device. Kim et al. developed three n-type hosts to form blue exciplex with mCBP (Kim et al., 2022). Among them, the exciplex developed by mCBP:CNmSi-2DBF-Trz showed a high T_1_ of 2.95 eV and the fabricated blue PhOLED showed a rather high EQE_max_ over 23%.

## Conclusion and Outlook

In summary, this study provided an overview of blue exciplex–based organic light-emitting materials and devices. The research background and luminescence mechanism were briefly introduced. Benefiting from the intriguing merits of exciplex-based OLEDs including low-driving voltages and low-efficiency roll-offs, simultaneously, as well as simple device structures, exciplexes have drawn significant attention on account of the potentials for efficient electroluminescence or for the use as high-performance cohost materials. Manipulating blue exciplex emissions by adjusting molecular structures gives an ideal strategy to fully utilize all exciton energies for high-performance OLEDs. We believe that our work will be conductive to the future development of blue exciplex–based OLEDs with high efficiencies and simplified device structures. Meanwhile, we expect that further systematic investigations of the excited-state dynamics and the structure-property relationships will be of benefit for the development of more efficient exciplex–based emitters and cohost materials oriented for WOLEDs and solution-processed OLEDs.

## References

[B1] Adachi C. Tsutsui T. Saito S. (1990). Blue Light‐emitting Organic Electroluminescent Devices. Appl. Phys. Lett. 56, 799–801. 10.1063/1.103177

[B2] Baldo M. A. O'Brien D. F. You Y. Shoustikov A. Sibley S. Thompson M. E. (1998). Highly Efficient Phosphorescent Emission from Organic Electroluminescent Devices. Nature 395, 151–154. 10.1038/25954

[B3] Ban X. Sun K. Sun Y. Huang B. Jiang W. (2016a). Enhanced Electron Affinity and Exciton Confinement in Exciplex-type Host: Power Efficient Solution-Processed Blue Phosphorescent Oleds with Low Turn-On Voltage. ACS Appl. Mat. Inter. 8, 2010–2016. 10.1021/acsami.5b10335 26726923

[B4] Ban X. X. Sun K. Y. Sun Y. M. Huang B. Jiang W. (2016b). Design of High Triplet Energy Electron Transporting Material for Exciplex-type Host: Efficient Blue and White Phosphorescent Oleds Based on Solution Processing. Org. Electron. 33, 9–14. 10.1016/j.orgel.2016.02.041

[B5] Ban X. X. Sun K. Y. Sun Y. M. Huang B. Ye S. H. Yang M. (2015). High Power Efficiency Solution-Processed Blue Phosphorescent Organic Light-Emitting Diodes Using Exciplex-type Host with a Turn-On Voltage Approaching the Theoretical Limit. ACS Appl. Mat. Inter. 7, 25129–25138. 10.1021/acsami.5b06424 26502064

[B6] Bernhard S. Gao X. Malliaras G. G. Abruña H. D. (2002). Efficient Electroluminescent Devices Based on a Chelated Osmium(ii) Complex. Adv. Mat. 14, 433–436. 10.1002/1521-4095(20020318)14:6<433::aid-adma433>3.0.co;2-w

[B7] Burroughes J. H. Bradley D. D. C. Brown A. R. Marks R. N. Mackay K. Friend R. H. (1990). Light-emitting-diodes Based on Conjugated Polymers. Nature 347, 539–541. 10.1038/347539a0

[B8] Chan C. Y. Tanaka M. Lee Y. T. Wong Y. W. Nakanotani H. Hatakeyama T. (2021). Stable Pure-Blue Hyperfluorescence Organic Light-Emitting Diodes with High-Efficiency and Narrow Emission. Nat. Photonics 15, 203–207. 10.1038/s41566-020-00745-z

[B9] Chang S. H. Chang C. F. Liao J. L. Chi Y. Zhou D. Y. Liao L. S. (2013). Emissive Osmium(ii) Complexes with Tetradentate Bis(pyridylpyrazolate) Chelates. Inorg. Chem. 52, 5867–5875. 10.1021/ic302829e 23621364

[B10] Chao C. Chen S. (1998). White Light Emission from Exciplex in a Bilayer Device with Two Blue Light-Emitting Polymers. Appl. Phys. Lett. 73, 426–428. 10.1063/1.121888

[B11] Chapran M. Pander P. Vasylieva M. Wiosna-Salyga G. Ulanski J. Dias F. B. (2019). Realizing 20% External Quantum Efficiency in Electroluminescence with Efficient Thermally Activated Delayed Fluorescence from an Exciplex. ACS Appl. Mat. Inter. 11, 13460–13471. 10.1021/acsami.8b18284 30864778

[B12] Chen Z. Liu X. K. Zheng C. J. Ye J. Liu C. L. Li F. (2015). High Performance Exciplex-Based Fluorescence-Phosphorescence White Organic Light-Emitting Device with Highly Simplified Structure. Chem. Mat. 27, 5206–5211. 10.1021/acs.chemmater.5b01188

[B13] Chou H. H. Cheng C. H. (2010). A Highly Efficient Universal Bipolar Host for Blue, Green, and Red Phosphorescent Oleds. Adv. Mat. 22, 2468–2471. 10.1002/adma.201000061 20446307

[B14] Cocchi M. Virgili D. Giro G. Fattori V. Di Marco P. Kalinowski J. (2002). Efficient Exciplex Emitting Organic Electroluminescent Devices. Appl. Phys. Lett. 80, 2401–2403. 10.1063/1.1467614

[B15] Du B. Wang X. Chen F. Yang Q. Shao S. Wang L. (2021). Through-space Charge Transfer Dendrimers Employing Oxygen-Bridged Triarylboron Acceptors for Efficient Deep-Blue Electroluminescence. Chem. Commun. 57, 7144–7147. 10.1039/d1cc02311j 34180922

[B16] Duan C. B. Han C. M. Du R. M. Wei Y. Xu H. (2018). High-efficiency Blue Dual-Emissive Exciplex Boosts Full-Radiative White Electroluminescence. Adv. Opt. Mat. 6, 1800437. 10.1002/Adom.201800437

[B17] Ehrmaier J. Rabe E. J. Pristash S. R. Corp K. L. Schlenker C. W. Sobolewski A. L. (2019). Singlet-triplet Inversion in Heptazine and in Polymeric Carbon Nitrides. J. Phys. Chem. A 123, 8099–8108. 10.1021/acs.jpca.9b06215 31466450

[B18] Endo A. Sato K. Yoshimura K. Kai T. Kawada A. Miyazaki H. (2011). Efficient Up-Conversion of Triplet Excitons into a Singlet State and its Application for Organic Light Emitting Diodes. Appl. Phys. Lett. 98, 083302. 10.1063/1.3558906

[B19] Friend R. H. Gymer R. W. Holmes A. B. Burroughes J. H. Marks R. N. Taliani C. (1999). Electroluminescence in Conjugated Polymers. Nature 397, 121–128. 10.1038/16393

[B20] Fukagawa H. Shimizu T. Ohbe N. Tokito S. Tokumaru K. Fujikake H. (2012). Anthracene Derivatives as Efficient Emitting Hosts for Blue Organic Light-Emitting Diodes Utilizing Triplet-Triplet Annihilation. Org. Electron. 13, 1197–1203. 10.1016/j.orgel.2012.03.019

[B21] Gebler D. D. Wang Y. Z. Blatchford J. W. Jessen S. W. Fu D. K. Swager T. M. (1997). Exciplex Emission in Bilayer Polymer Light-Emitting Devices. Appl. Phys. Lett. 70, 1644–1646. 10.1063/1.118657

[B22] Gould I. R. Farid S. Young R. H. (1992). Relationship between Exciplex Fluorescence and Electron Transfer in Radical Ion Pairs. J. Photochem. Photobiol. A 65, 133–147. 10.1016/1010-6030(92)85038-v

[B23] Goushi K. Adachi C. (2012). Efficient Organic Light-Emitting Diodes through Up-Conversion from Triplet to Singlet Excited States of Exciplexes. Appl. Phys. Lett. 101, 023306. 10.1063/1.4737006

[B24] Goushi K. Yoshida K. Sato K. Adachi C. (2012). Organic Light-Emitting Diodes Employing Efficient Reverse Intersystem Crossing for Triplet-To-Singlet State Conversion. Nat. Photonics 6, 253–258. 10.1038/nphoton.2012.31

[B25] Gu J. N. Tang Z. Y. Guo H. Q. Chen Y. Xiao J. Chen Z. J. (2022). Intermolecular Tadf: Bulk and Interface Exciplexes. J. Mat. Chem. C 10, 4521–4532. 10.1039/d1tc04950j

[B26] Guo J. F. Zhen Y. G. Dong H. L. Hu W. P. (2021). Recent Progress on Organic Exciplex Materials with Different Donor-Acceptor Contacting Modes for Luminescent Applications. J. Mat. Chem. C 9, 16843–16858. 10.1039/d1tc04330g

[B27] Guo Y. Y. Zhao Y. P. Miao Y. Q. Wang L. S. Li T. B. Wang H. (2020). All-exciplex-based White Organic Light-Emitting Diodes by Employing an Interface-free Sandwich Light-Emitting Unit Achieving High Electroluminescence Performance. J. Mat. Chem. C 8, 12247–12256. 10.1039/d0tc02915g

[B28] Hong G. Gan X. Leonhardt C. Zhang Z. Seibert J. Busch J. M. (2021). A Brief History of Oleds-Emitter Development and Industry Milestones. Adv. Mat. 33, e2005630. 10.1002/adma.202005630 33458866

[B29] Huang J. H. Su J. H. Tian H. (2012). The Development of Anthracene Derivatives for Organic Light-Emitting Diodes. J. Mat. Chem. 22, 10977–10989. 10.1039/c2jm16855c

[B30] Hung W. Y. Fang G. C. Lin S. W. Cheng S. H. Wong K. T. Kuo T. Y. (2014). The First Tandem, All-Exciplex-Based Woled. Sci. Rep. 4, 5161. 10.1038/srep05161 24895098 PMC4044637

[B31] Hung Y. T. Chen Z. Y. Hung W. Y. Chen D. G. Wong K. T. (2018). Exciplex Cohosts Employing Nonconjugated Linked Dicarbazole Donors for Highly Efficient Thermally Activated Delayed Fluorescence-Based Organic Light-Emitting Diodes. ACS Appl. Mat. Inter. 10, 34435–34442. 10.1021/acsami.8b14070 30222304

[B32] Jankus V. Chiang C. J. Dias F. Monkman A. P. (2013). Deep Blue Exciplex Organic Light-Emitting Diodes with Enhanced Efficiency; P-type or E-type Triplet Conversion to Singlet Excitons? Adv. Mat. 25, 1455–1459. 10.1002/adma.201203615 23281058

[B33] Jenekhe S. A. (1995). Excited-state Complexes of Conjugated Polymers. Adv. Mat. 7, 309–311. 10.1002/adma.19950070314

[B34] Jia J. L. Zhu L. P. Wei Y. Wu Z. B. Xu H. Ding D. X. (2015). Triazine-phosphine Oxide Electron Transporter for Ultralow-Voltage-Driven Sky Blue Pholeds. J. Mat. Chem. C 3, 4890–4902. 10.1039/c4tc02993c

[B35] Jung M. Lee J. Y. (2020). Exciplex Hosts for Blue Phosphorescent Organic Light-Emitting Diodes. J. Inf. Disp. 21, 11–18. 10.1080/15980316.2019.1680453

[B36] Kim H. B. Kim J. J. (2019). Recent Progress on Exciplex-Emitting Oleds. J. Inf. Disp. 20, 105–121. 10.1080/15980316.2019.1650838

[B37] Kim Y. S. Lim J. Lee J. Y. Lee Y. Choo C. (2022). Benzonitirile Modified N Type Host for Exciplex Host to Enhance Efficiency and Lifetime in Blue Phosphorescent Organic Light-Emitting Diodes. Chem. Eng. J. 429, 132584. 10.1016/j.cej.2021.132584

[B38] Kulkarni A. P. Jenekhe S. A. (2008). Blue-green, Orange, and White Organic Light-Emitting Diodes Based on Exciplex Electroluminescence of an Oligoquinoline Acceptor and Different Hole-Transport Materials. J. Phys. Chem. C 112, 5174–5184. 10.1021/jp076480z

[B39] Lee H. Karthik D. Lampande R. Ryu J. H. Kwon J. H. (2020). Recent Advancement in Boron-Based Efficient and Pure Blue Thermally Activated Delayed Fluorescence Materials for Organic Light-Emitting Diodes. Front. Chem. 8, 373. 10.3389/fchem.2020.00373 32509723 PMC7248410

[B40] Lee J. H. Cheng S. H. Yoo S. J. Shin H. Chang J. H. Wu C. I. (2015). An Exciplex Forming Host for Highly Efficient Blue Organic Light Emitting Diodes with Low Driving Voltage. Adv. Funct. Mat. 25, 361–366. 10.1002/adfm.201402707

[B41] Lee J. Lee J. I. Lee J. Y. Chu H. Y. (2009). Stable Efficiency Roll-Off in Blue Phosphorescent Organic Light-Emitting Diodes by Host Layer Engineering. Org. Electron. 10, 1529–1533. 10.1016/j.orgel.2009.08.020

[B42] Lee S. Kim K. H. Limbach D. Park Y. S. Kim J. J. (2013). Low Roll-Off and High Efficiency Orange Organic Light Emitting Diodes with Controlled Co-doping of Green and Red Phosphorescent Dopants in an Exciplex Forming Co-host. Adv. Funct. Mat. 23, 4105–4110. 10.1002/adfm.201300187

[B43] Li B. Yang Z. Gong W. Q. Chen X. H. Bruce D. W. Wang S. Y. (2021a). Intramolecular Through-Space Charge Transfer Based Tadf-Active Multifunctional Emitters for High Efficiency Solution-Processed Oled. Adv. Opt. Mat. 9, 2100180. 10.1002/Adom.202100180

[B44] Li J. Gong H. Zhang J. Liu H. Tao L. Wang Y. (2021b). Efficient Exciplex-Based Deep-Blue Organic Light-Emitting Diodes Employing a Bis(4-Fluorophenyl)amine-Substituted Heptazine Acceptor. Molecules 26, 5568. 10.3390/molecules26185568 34577041 PMC8466596

[B45] Li J. Gong H. Zhang J. Zhou S. Tao L. Jiang L. (2021c). Enhanced Electroluminescence Based on a Pi-Conjugated Heptazine Derivative by Exploiting Thermally Activated Delayed Fluorescence. Front. Chem. 9, 693813. 10.3389/fchem.2021.693813 34055753 PMC8155250

[B46] Li J. Li Z. Liu H. Gong H. Zhang J. Li X. (2022). Down-conversion-induced Delayed Fluorescence via an Inverted Singlet-Triplet Channel. Dyes Pigments 203, 110366. 10.1016/j.dyepig.2022.110366

[B47] Li J. Nakagawa T. MacDonald J. Zhang Q. Nomura H. Miyazaki H. (2013). Highly Efficient Organic Light-Emitting Diode Based on a Hidden Thermally Activated Delayed Fluorescence Channel in a Heptazine Derivative. Adv. Mat. 25, 3319–3323. 10.1002/adma.201300575 23670919

[B48] Li J. Nomura H. Miyazaki H. Adachi C. (2014). Highly Efficient Exciplex Organic Light-Emitting Diodes Incorporating a Heptazine Derivative as an Electron Acceptor. Chem. Commun. 50, 6174–6176. 10.1039/c4cc01590h 24781875

[B49] Li J. Zhang J. C. Gong H. Q. Tao L. Wang Y. Q. Guo Q. (2021d). Efficient Deep-Blue Electroluminescence Employing Heptazine-Based Thermally Activated Delayed Fluorescence. Photonics 8, 293. 10.3390/Photonics8080293

[B50] Lim H. Shin H. Kim K. H. Yoop S. J. Huh J. S. Kim J. J. (2017). An Exciplex Host for Deep-Blue Phosphorescent Organic Light-Emitting Diodes. ACS Appl. Mat. Inter. 9, 37883–37887. 10.1021/acsami.7b10914 28968060

[B51] Liu X. K. Chen W. Thachoth Chandran H. Qing J. Chen Z. Zhang X. H. (2016). High-performance, Simplified Fluorescence and Phosphorescence Hybrid White Organic Light-Emitting Devices Allowing Complete Triplet Harvesting. ACS Appl. Mat. Inter. 8, 26135–26142. 10.1021/acsami.6b07629 27608272

[B52] Liu X. K. Chen Z. Qing J. Zhang W. J. Wu B. Tam H. L. (2015a). Remanagement of Singlet and Triplet Excitons in Single-Emissive-Layer Hybrid White Organic Light-Emitting Devices Using Thermally Activated Delayed Fluorescent Blue Exciplex. Adv. Mat. 27, 7079–7085. 10.1002/adma.201502897 26436730

[B53] Liu X. K. Chen Z. Zheng C. J. Chen M. Liu W. Zhang X. H. (2015b). Nearly 100% Triplet Harvesting in Conventional Fluorescent Dopant-Based Organic Light-Emitting Devices through Energy Transfer from Exciplex. Adv. Mat. 27, 2025–2030. 10.1002/adma.201500013 25676085

[B54] Morteani A. Dhoot A. Friend R. (2003). Barrier-free Electron-Hole Capture in Polymer Blend Heterojunction Light-Emitting Diodes. Adv. Mat. 15, 1708–1712. 10.1002/adma.200305618

[B55] Nakanotani H. Higuchi T. Furukawa T. Masui K. Morimoto K. Numata M. (2014). High-efficiency Organic Light-Emitting Diodes with Fluorescent Emitters. Nat. Commun. 5, 4016. 10.1038/ncomms5016 24874292

[B56] Oh C. S. Kang Y. J. Jeon S. K. Lee J. Y. (2015). High Efficiency Exciplex Emitters Using Donor-Acceptor Type Acceptor Material. J. Phys. Chem. C 119, 22618–22624. 10.1021/acs.jpcc.5b05292

[B57] Osaheni J. A. Jenekhe S. A. (1994). Efficient Blue Luminescence of a Conjugated Polymer Exciplex. Macromolecules 27, 739–742. 10.1021/ma00081a018

[B58] Park Y. S. Kim K. H. Kim J. J. (2013a). Efficient Triplet Harvesting by Fluorescent Molecules through Exciplexes for High Efficiency Organic Light-Emitting Diodes. Appl. Phys. Lett. 102, 5. 10.1063/1.4802716

[B59] Park Y. S. Lee S. Kim K. H. Kim S. Y. Lee J. H. Kim J. J. (2013b). Exciplex-forming Co-host for Organic Light-Emitting Diodes with Ultimate Efficiency. Adv. Funct. Mat. 23, 4914–4920. 10.1002/adfm.201300547

[B60] Peng C. C. Yang S. Y. Li H. C. Xie G. H. Cui L. S. Zou S. N. (2020). Highly Efficient Thermally Activated Delayed Fluorescence via an Unconjugated Donor-Acceptor System Realizing Eqe of over 30. Adv. Mat. 32, 2003885. 10.1002/adma.202003885 33118645

[B61] Peng Q. Shuai Z. (2021). Molecular Mechanism of Aggregation‐induced Emission. Aggregate 2, e91. 10.1002/agt2.91

[B62] Peng Z. Bao Z. Galvin M. E. (1998). Oxadiazole-containing Conjugated Polymers for Light-Emitting Diodes. Adv. Mat. 10, 680–684. 10.1002/(sici)1521-4095(199806)10:9<680::aid-adma680>3.0.co;2-h

[B63] Pollice R. Friederich P. Lavigne C. Gomes G. D. Aspuru-Guzik A. (2021). Organic Molecules with Inverted Gaps between First Excited Singlet and Triplet States and Appreciable Fluorescence Rates. Matter 4, 1654–1682. 10.1016/j.matt.2021.02.017

[B64] Sarma M. Wong K. T. (2018). Exciplex: An Intermolecular Charge-Transfer Approach for Tadf. ACS Appl. Mat. Inter. 10, 19279–19304. 10.1021/acsami.7b18318 29613766

[B65] Shao J. Chen C. Zhao W. Zhang E. Ma W. Sun Y. (2022). Recent Advances of Interface Exciplex in Organic Light-Emitting Diodes. Micromachines 13, 298. 10.102903390/mi13020298 35208422 PMC8875368

[B66] Shao S. Wang L. (2020). Through‐space Charge Transfer Polymers for Solution‐processed Organic Light‐emitting Diodes. Aggregate 1, 45–56. 10.1002/agt2.4

[B67] Shin H. Lee S. Kim K. H. Moon C. K. Yoo S. J. Lee J. H. (2014). Blue Phosphorescent Organic Light-Emitting Diodes Using an Exciplex Forming Co-host with the External Quantum Efficiency of Theoretical Limit. Adv. Mat. 26, 4730–4734. 10.1002/adma.201400955 24838525

[B68] Stavrou K. Danos A. Hama T. Hatakeyama T. Monkman A. (2021). Hot Vibrational States in a High-Performance Multiple Resonance Emitter and the Effect of Excimer Quenching on Organic Light-Emitting Diodes. ACS Appl. Mat. Inter. 13, 8643–8655. 10.1021/acsami.0c20619 PMC8023512 33555850

[B69] Su S. J. Sasabe H. Takeda T. Kido J. (2008). Pyridine-containing Bipolar Host Materials for Highly Efficient Blue Phosphorescent Oleds. Chem. Mat. 20, 1691–1693. 10.1021/cm703682q

[B70] Suman G. R. Pandey M. Chakravarthy A. S. J. (2021). Review on New Horizons of Aggregation Induced Emission: From Design to Development. Mat. Chem. Front. 5, 1541–1584. 10.1039/d0qm00825g

[B71] Tan X. F. Volyniuk D. Matulaitis T. Keruckas J. Ivaniuk K. Helzhynskyy I. (2020). High Triplet Energy Materials for Efficient Exciplex-Based and Full-Tadf-Based White Oleds. Dyes Pigments 177, 108259. 10.1016/J.Dyepig.2020.108259

[B72] Tang C. W. Vanslyke S. A. Chen C. H. (1989). Electroluminescence of Doped Organic Thin-Films. J. Appl. Phys. 65, 3610–3616. 10.1063/1.343409

[B73] Tang C. W. VanSlyke S. A. (1987). Organic Electroluminescent Diodes. Appl. Phys. Lett. 51, 913–915. 10.1063/1.98799

[B74] Tang X. Cui L. S. Li H. C. Gillett A. J. Auras F. Qu Y. K. (2020). Highly Efficient Luminescence from Space-Confined Charge-Transfer Emitters. Nat. Mat. 19, 1332–1338. 10.1038/s41563-020-0710-z 32541938

[B75] Uoyama H. Goushi K. Shizu K. Nomura H. Adachi C. (2012). Highly Efficient Organic Light-Emitting Diodes from Delayed Fluorescence. Nature 492, 234–238. 10.1038/nature11687 23235877

[B76] Wang J. Kawabe Y. Peyghambarian N. (1998). Exciplex Electroluminescence from Organic Bilayer Devices Composed of Triphenyldiamine and Quinoxaline Derivatives. Adv. Mat. 10, 230–233. 10.1002/(sici)1521-4095(199802)10:3<230::aid-adma230>3.0.co;2-y

[B77] Wang Q. Tian Q. S. Zhang Y. L. Tang X. Liao L. S. (2019). High-efficiency Organic Light-Emitting Diodes with Exciplex Hosts. J. Mat. Chem. C 7, 11329–11360. 10.1039/c9tc03092a

[B78] Wang T. T. Xie G. H. Li H. C. Yang S. Y. Li H. Xiao Y. L. (2021a). Pi-stacked Thermally Activated Delayed Fluorescence Emitters with Alkyl Chain Modulation. CCS Chem. 3, 1757–1763. 10.31635/ccschem.020.202000355

[B79] Wang X. Hu J. Lv J. Yang Q. Tian H. Shao S. (2021b). Pi-stacked Donor-Acceptor Dendrimers for Highly Efficient White Electroluminescence. Angew. Chem. Int. Ed. 60, 16585–16593. 10.1002/anie.202104145 33942454

[B80] Wu X. G. Su B. K. Chen D. G. Liu D. H. Wu C. C. Huang Z. X. (2021). The Role of Host-Guest Interactions in Organic Emitters Employing Mr-Tadf. Nat. Photonics 15, 780–786. 10.1038/s41566-021-00870-3

[B81] Wu Z. B. Yu L. Zhao F. C. Qiao X. F. Chen J. S. Ni F. (2017). Precise Exciton Allocation for Highly Efficient White Organic Light-Emitting Diodes with Low Efficiency Roll-Off Based on Blue Thermally Activated Delayed Fluorescent Exciplex Emission. Adv. Opt. Mat. 5, 1700415. 10.1002/adom.201700415

[B82] Xiao P. Huang J. H. Yu Y. C. Yuan J. Luo D. X. Liu B. Q. (2018). Recent Advances of Exciplex-Based White Organic Light-Emitting Diodes. Appl. Sci. 8, 1449. 10.3390/App8091449

[B83] Xue Q. Xie G. H. (2021). Thermally Activated Delayed Fluorescence beyond Through-Bond Charge Transfer for High-Performance Oleds. Adv. Opt. Mat. 9, 2002204. 10.1002/Adom.202002204

[B84] Yang C. C. Hsu C. J. Chou P. T. Cheng H. C. Su Y. O. Leung M. K. (2010). Excited State Luminescence of Multi-(5-Phenyl-1,3,4-Oxadiazo-2-Yl)benzenes in an Electron-Donating Matrix: Exciplex or Electroplex? J. Phys. Chem. B 114, 756–768. 10.1021/jp9093063 20039615

[B85] Yang S. Y. Wang Y. K. Peng C. C. Wu Z. G. Yuan S. Yu Y. J. (2020). Circularly Polarized Thermally Activated Delayed Fluorescence Emitters in Through-Space Charge Transfer on Asymmetric Spiro Skeletons. J. Am. Chem. Soc. 142, 17756–17765. 10.1021/jacs.0c08980 33021373

[B86] Yun J. H. Lee K. H. Chung W. J. Lee J. Y. Lee Y. Lyu J. J. (2021a). Thermally Activated Delayed Fluorescence Type Exciplex Host for Long Lifetime in Deep Blue Phosphorescent Organic Light-Emitting Diodes. Chem. Eng. J. 417, 128086. 10.1016/j.cej.2020.128086

[B87] Yun J. H. Lim J. Lee J. Y. Lee Y. Chu C. (2021b). Triplet Exciton Upconverting Blue Exciplex Host for Deep Blue Phosphors. Chem-Eur. J. 27, 12642–12648. 10.1002/chem.202101819 34240481

[B88] Zhang M. Zheng C. J. Lin H. Tao S. L. (2021). Thermally Activated Delayed Fluorescence Exciplex Emitters for High-Performance Organic Light-Emitting Diodes. Mat. Horiz. 8, 401–425. 10.1039/d0mh01245a 34821262

[B89] Zhang Q. S. Li J. Shizu K. Huang S. P. Hirata S. Miyazaki H. (2012). Design of Efficient Thermally Activated Delayed Fluorescence Materials for Pure Blue Organic Light Emitting Diodes. J. Am. Chem. Soc. 134, 14706–14709. 10.1021/ja306538w 22931361

[B90] Zhang T. Zhao B. Chu B. Li W. Su Z. Yan X. (2015). Simple Structured Hybrid Woleds Based on Incomplete Energy Transfer Mechanism: From Blue Exciplex to Orange Dopant. Sci. Rep. 5, 10234. 10.1038/srep10234 25975371 PMC4432569

[B91] Zhao B. Zhang T. Y. Chu B. Li W. L. Su Z. S. Luo Y. S. (2015). Highly Efficient Tandem Full Exciplex Orange and Warm White Oleds Based on Thermally Activated Delayed Fluorescence Mechanism. Org. Electron. 17, 15–21. 10.1016/j.orgel.2014.11.014

[B92] Zhao C. Y. Yan D. H. Ahamad T. Alshehri S. M. Ma D. G. (2019). High Efficiency and Low Roll-Off Hybrid White Organic Light Emitting Diodes by Strategically Introducing Multi-Ultrathin Phosphorescent Layers in Blue Exciplex Emitter. J. Appl. Phys. 125, 045501. 10.1063/1.5047861

[B93] Zhao D. W. Zhang F. J. Xu C. Sun J. Y. Song S. F. Xu Z. (2008). Exciplex Emission in the Blend of Two Blue Luminescent Materials. Appl. Surf. Sci. 254, 3548–3552. 10.1016/j.apsusc.2007.11.049

[B94] Zhou L. Kwong C. L. Kwok C. C. Cheng G. Zhang H. Che C. M. (2014). Efficient Red Electroluminescent Devices with Sterically Hindered Phosphorescent Platinum(ii) Schiff Base Complexes and Iridium Complex Codopant. Chem. Asian J. 9, 2984–2994. 10.1002/asia.201402618 25145872

[B95] Zhu J. Z. Li W. L. Han L. L. Chu B. Zhang G. Yang D. F. (2009). Very Broad White-Emission Spectrum Based Organic Light-Emitting Diodes by Four Exciplex Emission Bands. Opt. Lett. 34, 2946–2948. 10.1364/ol.34.002946 19794777

[B96] Zou Y. Hu J. Yu M. Miao J. Xie Z. Qiu Y. (2022). High Performance Narrowband Pure‐red Oleds with External Quantum Efficiencies up to 36.1% and Ultra‐low Efficiency Roll‐off. Adv. Mat. 19, e2201442. 10.1002/adma.202201442 35588162

